# Patient-derived PixelPrint phantoms for evaluating clinical imaging performance of a deep learning CT reconstruction algorithm

**DOI:** 10.1088/1361-6560/ad3dba

**Published:** 2024-05-14

**Authors:** Jessica Y Im, Sandra S Halliburton, Kai Mei, Amy E Perkins, Eddy Wong, Leonid Roshkovan, Olivia F Sandvold, Leening P Liu, Grace J Gang, Peter B Noël

**Affiliations:** 1 Department of Radiology, University of Pennsylvania, Philadelphia, PA, United States of America; 2 Department of Bioengineering, University of Pennsylvania, Philadelphia, PA, United States of America; 3 Philips Healthcare, Cleveland, OH, United States of America

**Keywords:** deep learning reconstruction, CT imaging phantoms, dose reduction, image quality assessment, 3D-printing, 3D-printed phantom

## Abstract

*Objective*. Deep learning reconstruction (DLR) algorithms exhibit object-dependent resolution and noise performance. Thus, traditional geometric CT phantoms cannot fully capture the clinical imaging performance of DLR. This study uses a patient-derived 3D-printed PixelPrint lung phantom to evaluate a commercial DLR algorithm across a wide range of radiation dose levels. *Method*. The lung phantom used in this study is based on a patient chest CT scan containing ground glass opacities and was fabricated using PixelPrint 3D-printing technology. The phantom was placed inside two different size extension rings to mimic a small- and medium-sized patient and was scanned on a conventional CT scanner at exposures between 0.5 and 20 mGy. Each scan was reconstructed using filtered back projection (FBP), iterative reconstruction, and DLR at five levels of denoising. Image noise, contrast to noise ratio (CNR), root mean squared error, structural similarity index (SSIM), and multi-scale SSIM (MS SSIM) were calculated for each image. *Results.* DLR demonstrated superior performance compared to FBP and iterative reconstruction for all measured metrics in both phantom sizes, with better performance for more aggressive denoising levels. DLR was estimated to reduce dose by 25%–83% in the small phantom and by 50%–83% in the medium phantom without decreasing image quality for any of the metrics measured in this study. These dose reduction estimates are more conservative compared to the estimates obtained when only considering noise and CNR. *Conclusion*. DLR has the capability of producing diagnostic image quality at up to 83% lower radiation dose, which can improve the clinical utility and viability of lower dose CT scans. Furthermore, the PixelPrint phantom used in this study offers an improved testing environment with more realistic tissue structures compared to traditional CT phantoms, allowing for structure-based image quality evaluation beyond noise and contrast-based assessments.

## 1. Introduction

Over the last few years, there has been substantial interest in the development and clinical use of deep learning reconstruction (DLR) algorithms for improving computed tomography (CT) image quality and reducing radiation dose (Koetzier *et al* 2023). For decades, filtered back projection (FBP) was the dominant reconstruction algorithm due to its numerical stability and fast computation time (Willemink and Noël 2019). However, at lower doses, FBP image quality drops and image noise increases dramatically (Koetzier *et al* 2023). With continued interest in dose reduction (Brenner and Hall 2007), especially in pediatric populations (Miglioretti *et al* 2013, Nagayama *et al* 2021, Sun *et al* 2021, Son *et al* 2022), clinical CT imaging has begun moving away from FBP toward newer solutions such as iterative reconstruction (IR) which preserves image quality at lower doses. Various forms of IR have demonstrated significant potential to minimize noise and thus to reduce dose compared to FBP (Willemink *et al* 2013). However, limitations in IR including unnatural noise texture (Willemink *et al* 2013, Philips Healthcare 2024) and extended reconstruction time (Willemink *et al* 2013, Koetzier *et al* 2023) have resulted in a push for further innovation in reconstruction solutions.

DLR for CT has emerged as a novel solution for improving image quality and reconstruction time while preserving FBP-like noise textures. These algorithms utilize artificial neural networks such as convolutional neural networks (CNNs) (Kang *et al* 2017, Chen *et al* 2017) or generative adversarial networks (GANs) (Wolterink *et al* 2017) which are trained to produce optimized output images from lower dose input data. DLR frameworks can be broadly categorized as either indirect, where a deep learning network is used alongside FBP or IR, or direct, in which the network directly converts sinogram data to image data without FBP or IR (Koetzier *et al* 2023). Many different implementations of DLR have been proposed in academic research (Wu *et al* 2017, Yang *et al* 2018, Bao *et al* 2019) as well as introduced clinically by CT vendors (Hsieh *et al* 2019, Boedeker 2021, Philips Healthcare 2024).

With the rise of commercially available DLR algorithms, there has been an increase in studies evaluating DLR. Multiple patient and phantom studies have demonstrated that DLR can improve image quality at low doses through enhanced lesion detectability and reduced noise (Akagi *et al* 2019, Nakamura *et al* 2019, Nagayama *et al* 2021, Sun *et al* 2021, Greffier *et al* 2022a, Miyata *et al* 2022, Park *et al* 2022, Greffier *et al* 2022b, Mikayama *et al* 2022, Son *et al* 2022, Greffier *et al* 2023a). These studies utilize quantitative metrics such as signal-to-noise ratio (SNR), contrast-to-noise ratio (CNR), noise, detectability index (d’), and noise power spectrum (NPS). In addition, qualitative scores for various aspects of subjective image quality have been obtained via reader studies by experienced radiologists. The literature has shown that various implementations of DLR can reduce dose by about 30%–71% compared to hybrid iterative reconstruction (HIR) methods while preserving diagnostic image quality (Koetzier *et al* 2023).

While there are many promising results regarding DLR performance, there are several limitations to current studies. First, due to the nonlinear nature of DLR, images reconstructed with DLR demonstrate object-dependent resolution and noise (Li *et al* 2022, Solomon *et al* 2020, Higaki *et al* 2020, Greffier *et al* 2023b). Traditional CT phantoms used in DLR evaluation studies are often composed of simple geometric shapes which are not designed to represent realistic tissue structures (Greffier *et al* 2022a, 2022b, Mikayama *et al* 2022). As a result, general image quality metrics such as noise and CNR measured on traditional CT phantoms cannot fully capture the clinical imaging performance of DLR (Samei *et al* 2019). Second, clinical imaging studies using patient data are often limited by sample size and restricted by radiation dose exposure concerns (Akagi *et al* 2019, Greffier *et al* 2023a, Lyu *et al* 2023), which limit the acceptable dose range as well as the number of times a patient can be scanned. Furthermore, patient scans do not have reliable ground truth images for comparison and thus cannot be used to assess the structural accuracy of a reconstructed image. A clinical scenario in which structural accuracy is important is lung CT imaging with ground glass opacity (GGO) findings. Subtle differences in shape (round versus polygonal, with or without radial growths) and texture (presence or absence of solid densities) in a GGO can lead to differences in image interpretation and clinical decision making (Infante *et al* 2009). Because of this, the accurate reconstruction of such structures and details is critical to ensuring the highest quality of patient care. Previous studies have investigated the general image quality and detectability of reconstructed structures but have not directly addressed the question of reconstruction accuracy of complex structures and textures such as those found in GGOs. Image reconstruction for clinical scenarios such as this require evaluation beyond what is currently available with phantom and patient studies.

This study proposes to use a patient-derived PixelPrint (Mei *et al* 2022a, Shapira *et al* 2023, 2022) phantom as a novel solution to address the current limitations in the evaluation of DLR performance. PixelPrint is a technology which produces 3D-printed patient-based phantoms which demonstrate highly detailed tissue structures, realistic textures, and accurate attenuation profiles. PixelPrint software converts 3D CT images into geometric code (g-code) instructions for fused filament fabrication (FFF) 3D printers by taking advantage of the partial volume effect to produce desired Hounsfield Unit (HU) values (Shapira *et al* 2022). Previous studies have demonstrated a high degree of HU and geometric similarity between scans of PixelPrint phantoms and their reference patient scans (Mei *et al* 2022b). Furthermore, reader studies demonstrated that there was no clinically significant difference in image quality assessment between reading a phantom lung image and reading a patient lung image (Shapira *et al* 2023). Compared to standard geometric CT imaging phantoms, PixelPrint phantoms demonstrate realistic tissue morphology and thus can more fully capture the clinical imaging performance of DLR. Compared to patient data, PixelPrint phantoms allow for more flexibility in radiation dose usage and have more accurate ground truth images with which to assess the structural precision of DLR images.

This study utilized a 3D-printed PixelPrint lung phantom to evaluate the clinical imaging performance of a commercial DLR algorithm, precise Image (PI) (Philips Healthcare, Cleveland, OH, USA) (Philips Healthcare 2024) compared to FBP and IR, with particular focus on the question of structural accuracy of reconstructed anatomy. PI is an example of a direct DLR algorithm and utilizes simulated low dose sinogram data for CNN training (Koetzier *et al* 2023, Philips Healthcare 2024). The PixelPrint phantom was scanned with a large range of radiation doses to investigate the dose reduction potential of each algorithm. As image quality is affected by patient size (i.e. CT images of large patients tend to have higher noise and reduced image quality compared to smaller patients), two different phantom sizes were included in the performance assessment to examine the generalizability of results to different patient sizes.

## 2. Methods

### 2.1. Patient CT scan selection

**Figure 1.**
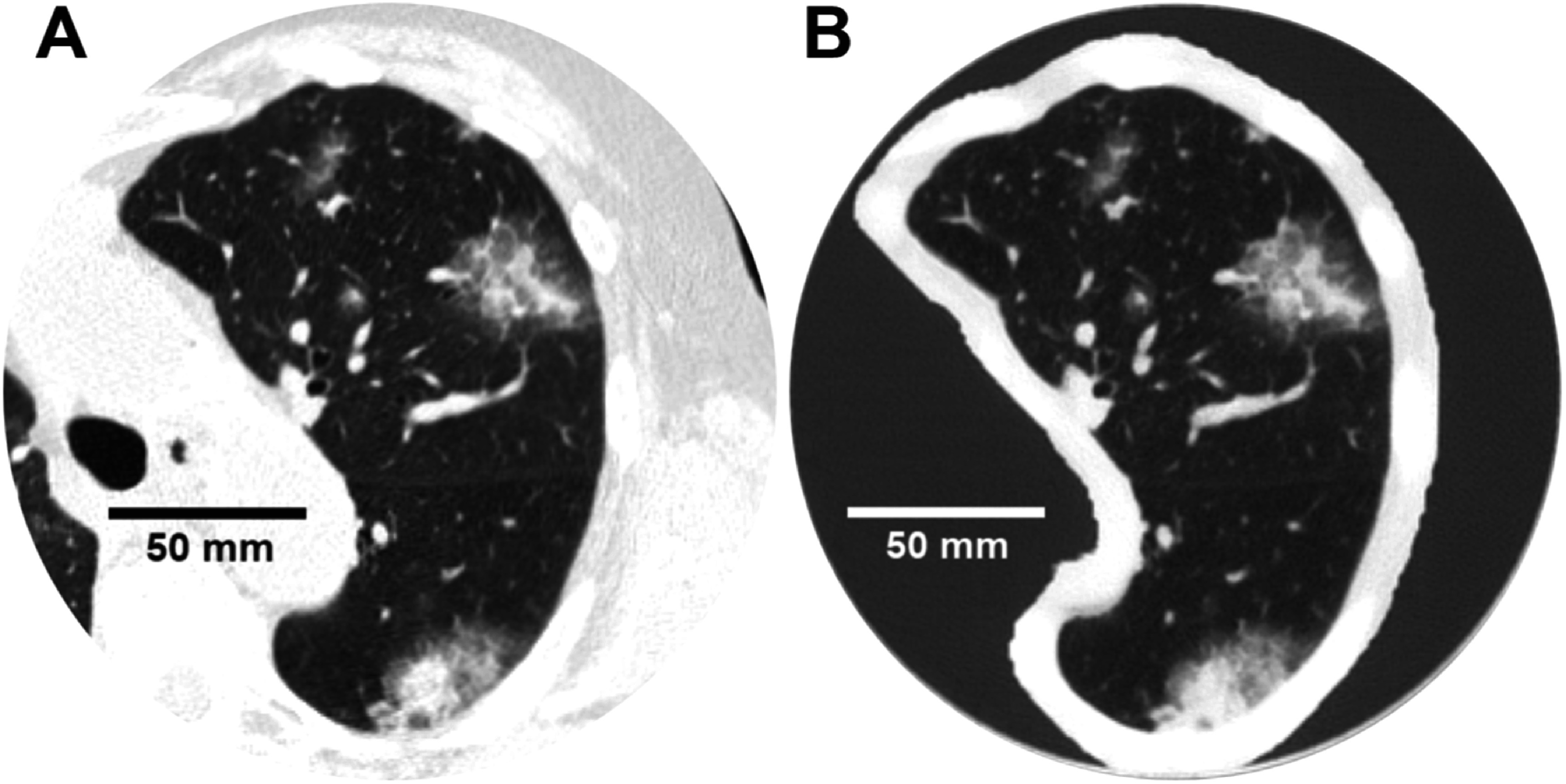
(A) Image of the left lung from the patient chest CT scan which was used to generate the phantom. (B) CT scan of the printed phantom, scanned at 20 mGy and 120 kVp and reconstructed using PI-Sharp. WL: −500, WW: 1100 HU.

The institutional review board (IRB) at the University of Pennsylvania approved this retrospective study (IRB Protocol #853697). A single patient chest CT scan containing multiple subsolid GGOs representing metastatic lesions was retrospectively selected as the model for the 3D-printed phantom in this study (figure 1). The image was taken from the Hospital of the University of Pennsylvania PACS system and anonymized. GGO lesions are an example of highly detailed lung structures in which accurate reconstruction of textures and shapes is clinically important. The scan and reconstruction parameters of the patient CT scan are listed in table 1.

**Table 1.** Scan and reconstruction parameters of the patient CT image.

Scanner model	Philips spectral CT 7500
Scan mode	Helical
Tube voltage	120 kVp
Tube current	173 mA
Rotation time	0.4 s
Helical pitch	1.15
Exposure	60 mAs
CTDI_vol_	4.7 mGy
Collimation	128 × 0.625 mm
Slice thickness	1 mm
Slice increment	1 mm
Reconstruction filter	YA
Reconstructed field of view	368 × 368 mm^2^
Matrix size	512 × 512 pixel^2^
Pixel spacing	0.7188 mm

### 2.2. Phantom fabrication

The phantom was fabricated using PixelPrint technology (Shapira *et al* 2022, 2023) to produce a realistic patient-specific lung CT phantom. The entire phantom was 3D-printed as one piece using polylactic acid (PLA) filament on an FFF printer (Lulzbot TAZ Sidekick with M175 v2 tool head, Fargo Additive Manufacturing Equipment 3D, LLC Fargo, ND, USA). The phantom was designed as a 20 cm diameter cylinder containing the segmented left lung positioned in the center of the cylinder. A 4 cm scan length containing a large (4.5 × 3.2 cm^2^) GGO was selected. The left lung was segmented using an open-source automated U-net lung segmentation model (Hofmanninger *et al* 2020). A 1 cm border of tissue surrounding the lung including parts of the ribs, thoracic muscles, and mediastinum was also included in the phantom. The regions inside of the segmented lung and border were printed using PixelPrint technology to modulate density and accurately reproduce the HU profiles of the patient image, within the HU range attainable with PLA (−867 to 115 HU) (Mei *et al* 2022b). Regions of the cylinder outside of the border were printed with a constant infill ratio of 15% (corresponding to ∼ −800 HU).

### 2.3. Image acquisition and reconstruction

The phantom was scanned with a default high resolution chest imaging protocol on a conventional CT scanner (Incisive CT, Philips Healthcare, Cleveland, OH, USA). Multiple scans were acquired with varying radiation dose levels ranging from 0.5 to 20 mGy. Scans were repeated three times at each dose level and each scan was reconstructed using FBP, an iterative reconstruction algorithm (iDose (Miglioretti *et al* 2013)) at a single noise level (Level 3), and DLR (PI) at five levels with increasingly aggressive noise reduction (Sharper, Sharp, Standard, Smooth, Smoother) (table 2). Additional scan and reconstructions parameters common to all scans are listed in table 3.

**Table 2.** Varying radiation dose levels used for phantom scanning and the different methods used for reconstruction.

Exposure [mAs]	CTDI_vol_ [mGy]	Reconstruction algorithms
250^a^	20^a^	FBP, YC Filter^a^
235	19	
185	15	iDose^4^ Level 3, YC filter
148^b^	12^b^	Precise Image (PI), Lung Definition, Sharper/Sharp/Standard/Smooth/Smoother
111^c^	9^c^	
74	6	
49	4	
25^d^	2^d^	
12	1	
6	0.5	

^a^ Dose and reconstruction parameters used for ground truth image.

^b^ Diagnostic reference level for a 29–33 cm water-equivalent diameter patient (Radiology ACo 2018).

^c^ Achievable dose level for a 29–33 cm water-equivalent diameter patient (Radiology ACo 2018).

^d^ Lung cancer screening level (Kazerooni *et al* 2014).

**Table 3.** CT scan and reconstruction parameters for the phantom scans.

Scanner model	Philips incisive CT
Scan mode	Helical
Tube voltage	120 kVp
Rotation time	0.5 s
Helical pitch	1
Collimation	64 × 0.625 mm
Slice thickness	1 mm
Slice increment	0.5 mm
Reconstructed field of view	350 × 350 mm^2^
Matrix size	512 × 512 pixel^2^
Pixel spacing	0.6836 mm

### 2.4. Extension rings

**Figure 2.**
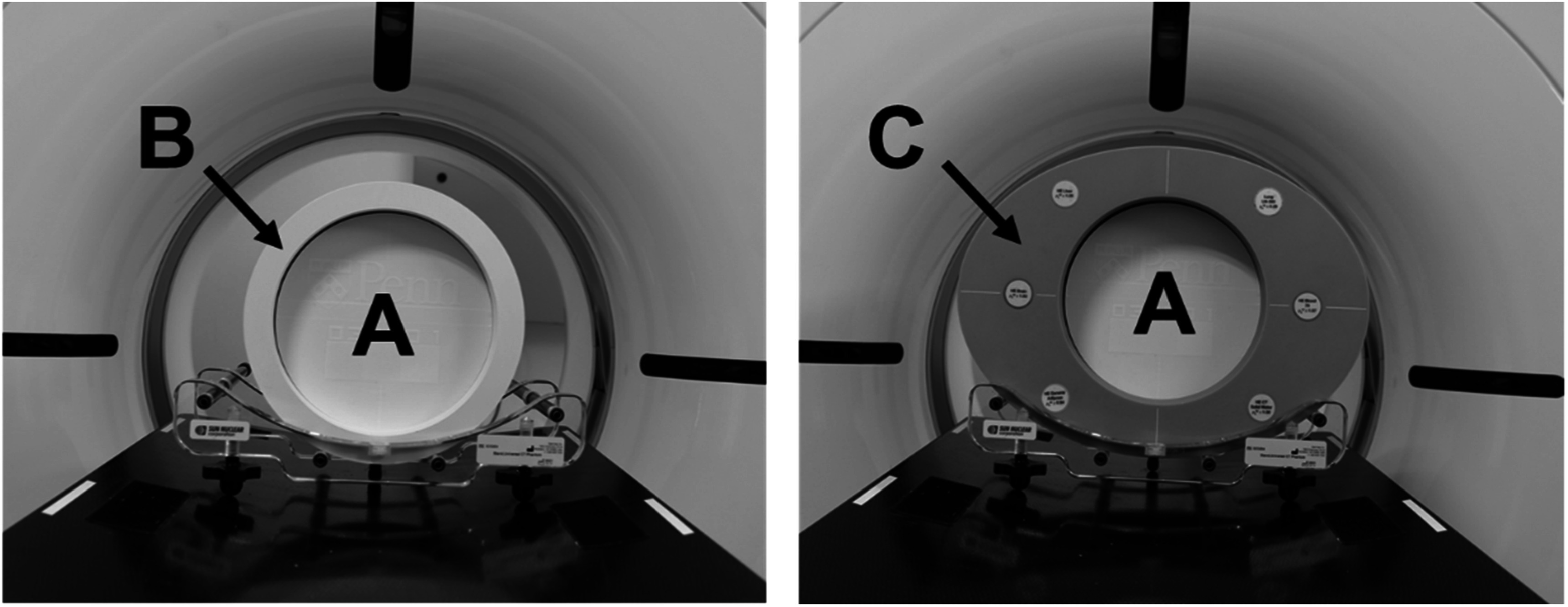
The PixelPrint lung phantom (A) placed inside a 25 × 25 cm^2^ 3D printed extension ring (B) to represent a small-sized patient and placed inside of a 30 × 40 cm^2^ MECT extension ring (C) to represent a medium-sized patient.

Patient size has a significant impact on the noise and image quality of CT images and, thus, can affect the performance of DLR. To mimic different patient sizes, the lung phantom was placed inside two different size extension rings during scanning (figure 2). A custom 25 × 25 cm^2^ water-equivalent extension ring with a 20 cm cylindrical bore was 3D printed using PLA filament. The lung phantom was placed in this custom extension ring to represent a small-sized patient (small phantom), resulting in a total water equivalent diameter (*D*_w_) of about 19 cm. To represent a medium-sized patient, the lung phantom was placed in the 20 cm bore of a 30 × 40 cm^2^ multi-energy CT phantom (MECT) (Sun Nuclear, WI, USA) extension ring (medium phantom). The *D*_w_ of the phantom plus MECT extension ring was about 30 cm. The small and medium phantom *D*_w_’s are representative of patient *D*_w_’s of an average pediatric (McCollough *et al* 2022) and adult (Kanal *et al* 2017) chest, respectively. The scan and reconstruction parameters outlined in tables 2 and 3 were repeated for each phantom size.

### 2.5. Image analysis

Image noise and CNR were calculated for each reconstruction and dose combination. The image noise was calculated for a 2 × 2 cm^2^ region of interest (ROI) across 10 consecutive slices in a homogeneous region of the phantom background lung parenchyma. The CNR was calculated between the GGO lesion and the background lung parenchyma where the GGO ROI was a 2 × 2 cm^2^ ROI over 14 consecutive slices inside of the GGO lesion. The equations used for noise and CNR calculations were:

\begin{eqnarray*}\mathrm{Noise}={\sigma }_{\mathrm{Background}}\end{eqnarray*}

\begin{eqnarray*}\mathrm{CNR}=\displaystyle \frac{{\mu }_{\mathrm{GGO}}-{\mu }_{\mathrm{Background}}}{{\sigma }_{\mathrm{Background}}},\end{eqnarray*}

where ${\sigma }_{\mathrm{Background}}$ is the standard deviation of HU values in the background lung ROI, ${\mu }_{\mathrm{GGO}}$ is the mean HU in the GGO ROI, and ${\mu }_{\mathrm{Background}}$ is the mean HU in the background lung ROI.

**Figure 3.**
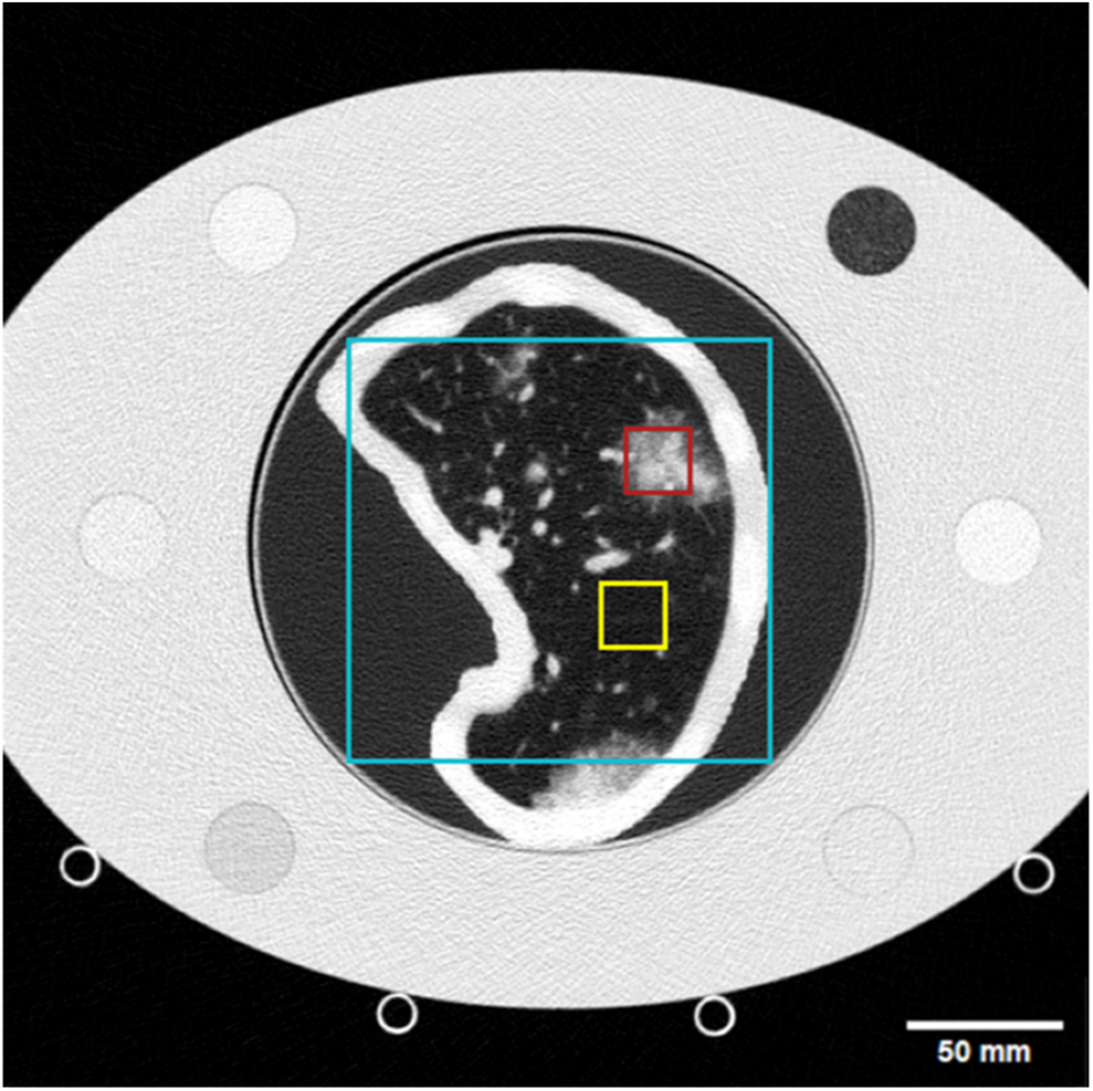
CT scan of the phantom placed inside of the medium size extension ring with marked ROIs used for image quality metric measurements. The yellow box encompasses the background lung ROI used for image noise and CNR calculations, the red box shows the GGO ROI used to calculate CNR, and the cyan box represents the ROI used for RMSE, SSIM, and MS SSIM measurements. WL: −450, WW: 1100 HU.

In addition to these general image quality metrics, the structural accuracy of the reconstructed images was evaluated using the image similarity metrics: root mean squared error (RMSE), structural similarity index measure (SSIM) (Wang *et al* 2004), and multi-scale SSIM (MS SSIM) (Wang *et al* 2003), using the highest dose (20 mGy) FBP image as the ground truth image. RMSE provides a direct intensity-based comparison between the reconstructed image and the ground truth, while SSIM takes luminance, contrast, and structural features into consideration. MS SSIM further expands on SSIM by generalizing the SSIM algorithm to incorporate image information at a variety of different resolution scales. The use of several similarity metrics helps ensure that the results are robust to different methods of assessing how closely the reconstructed images match the ground truth. All similarity metrics were measured in a 13.5 × 13.5 cm^2^ ROI across 50 consecutive slices within the 3D printed phantom. The RMSE and SSIM were calculated for each image using the open source Python package skimage.metrics (van der Walt *et al* 2014), and the MS SSIM was calculated using the open source python library pytorch-msssim (Pytorch-msssim 2023). All ROIs used for these calculations are shown in figure 3, and the same ROIs were used for each of the reconstructed images. Since the 20 mGy scans were used as the ground truth, they were excluded from the sample for all image metric calculations.

### 2.6. Statistical analysis

The performance of each dose and reconstruction combination was evaluated in comparison to the performance of the FBP images of scans taken at 12 mGy, which is the diagnostic reference level for 29–33 cm water equivalent diameter patients (Radiology ACo 2018). A two-sample, one-tailed t-test was performed for each image metric using the open-source python package Scipy statistical functions (Virtanen *et al* 2020). Effects were considered statistically significant where $p< 0.05,$ which after applying the Bonferroni *post hoc* correction results in $p< \tfrac{0.05}{\#\,\mathrm{metrics}}< \tfrac{0.05}{5}< 0.01.$ The potential dose reduction of each reconstruction algorithm was then determined by finding the lowest dose measured at which there was no statistically significant decrease in image quality from the reference for any measured metric.

## 3. Results

### 3.1. Comparison of reconstruction algorithms

**Figure 4.**
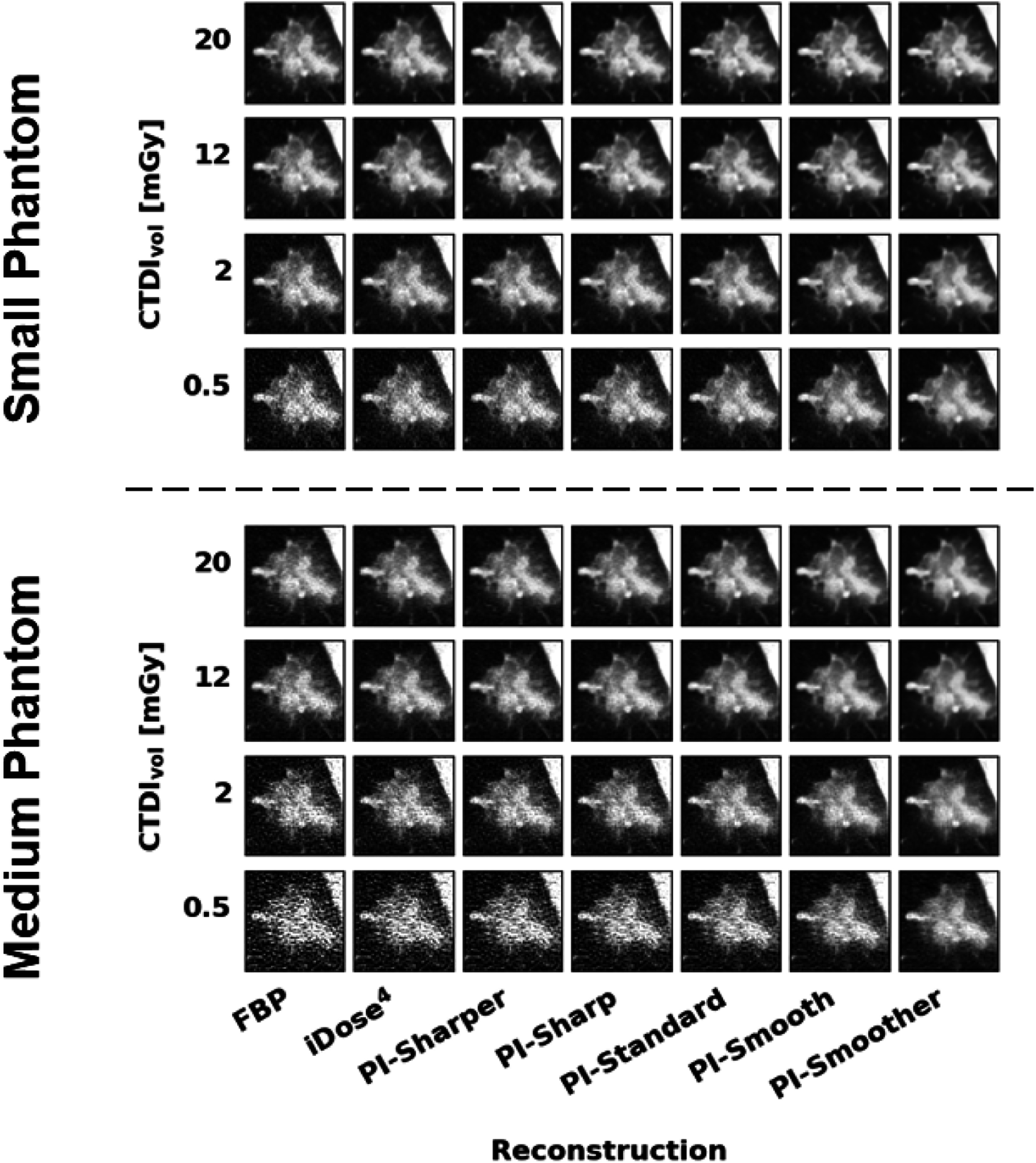
Images of the GGO lesion taken from the small phantom (top) and medium phantom (bottom) at several dose and reconstruction combinations. Figures A1 and A2 in the appendix show the GGO lesion images from all the dose levels collected. WL: −500, WW: 1000 HU.

**Figure 5.**
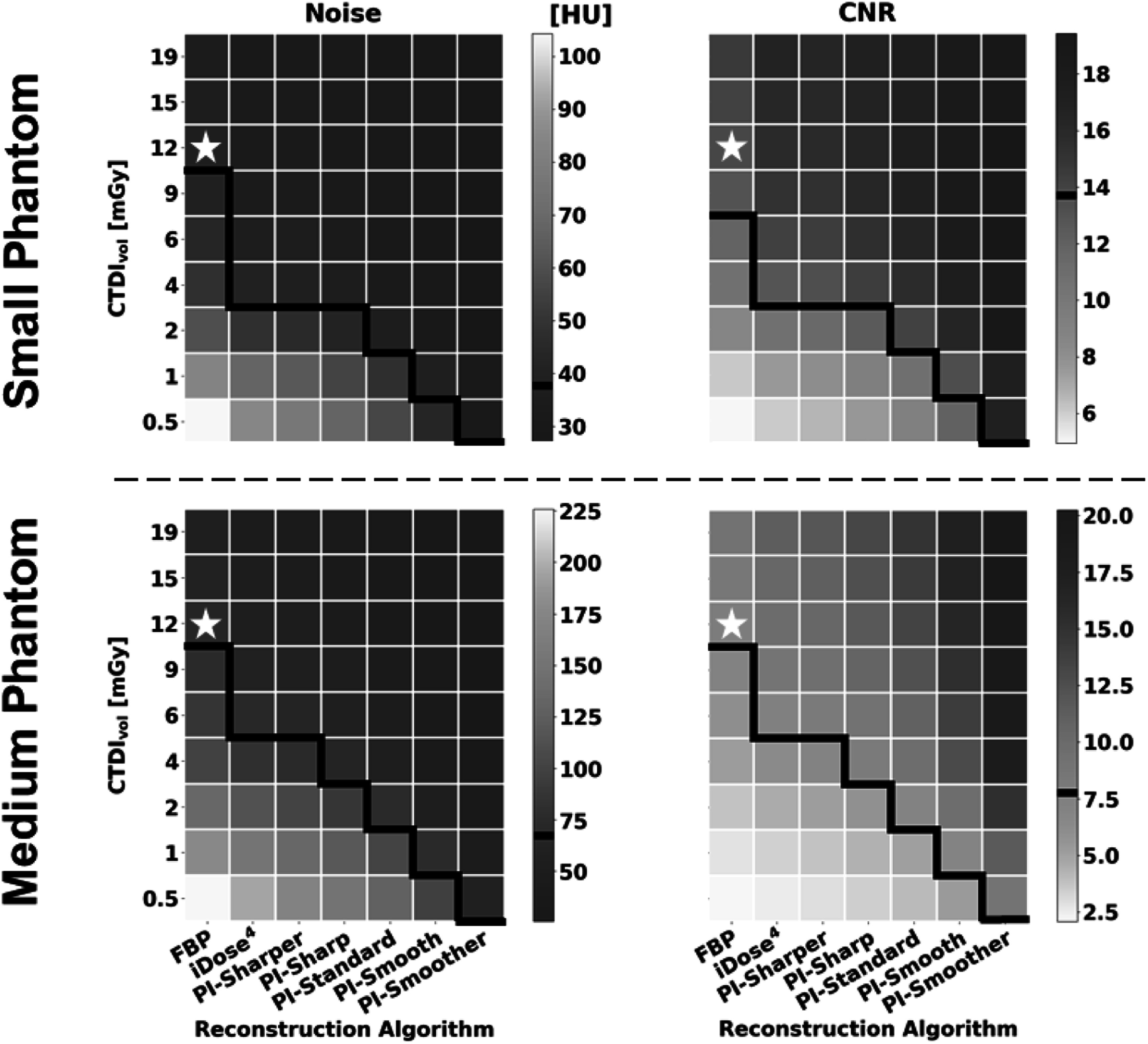
Heatmaps displaying results of the image metric calculations for Noise (left) and CNR (right) from both the small phantom (top) and medium phantom (bottom). A white star is used to designate the dose and reconstruction combination used as the reference for statistical comparison for each metric. The value for this reference group is indicated on the color bars by a black line. The black lines on the heatmaps separate values that are statistically better than or equivalent to the reference (above the line) from those that are statistically worse than the reference (below the line).

**Figure 6.**
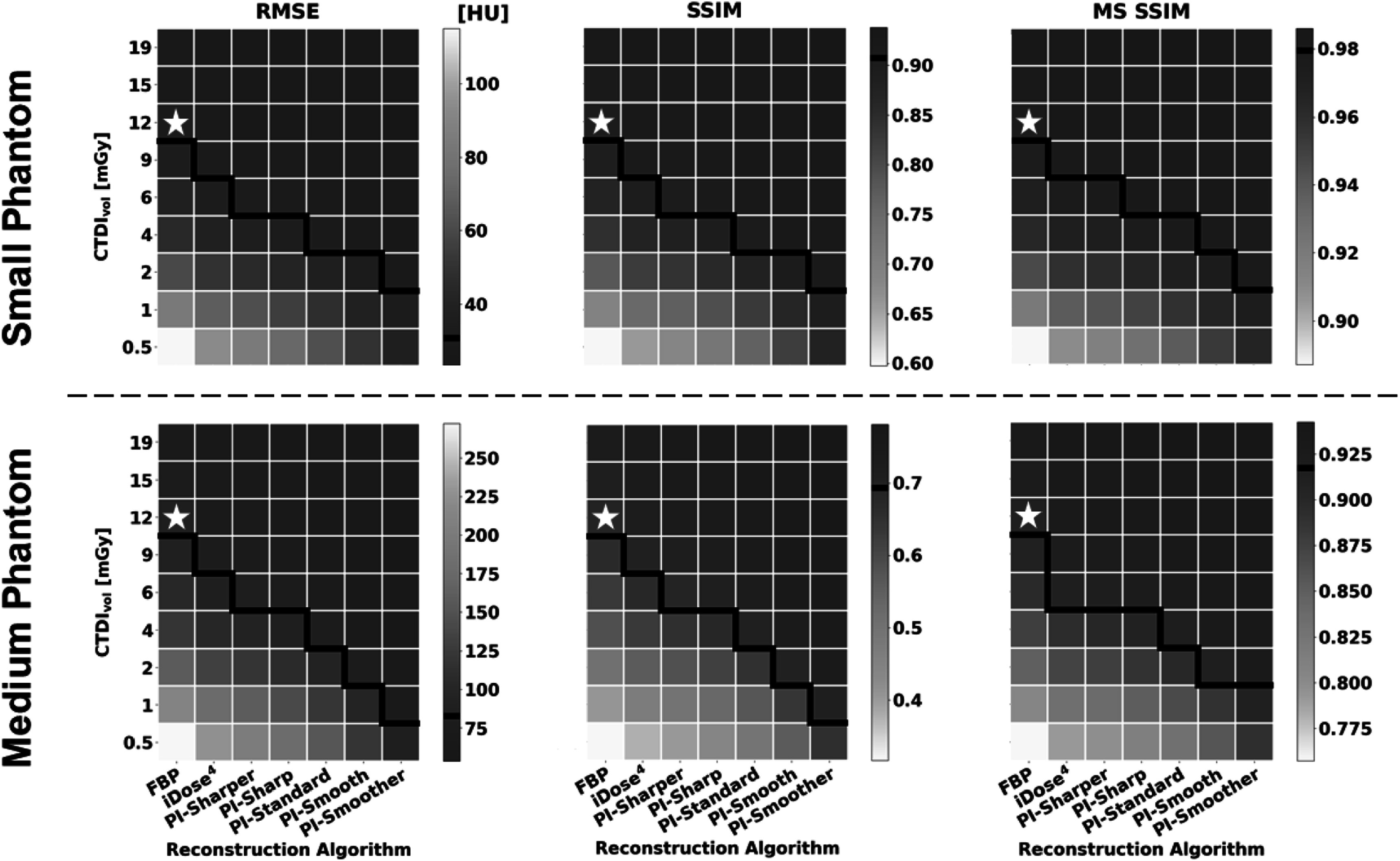
Heatmaps displaying results of the image metric calculations for RMSE (left), SSIM (middle), and MS SSIM (right) from both the small phantom (top) and medium phantom (bottom). The white stars and black lines in this figure have the same function as in figure 5.

PI demonstrates superior performance compared to both FBP and iterative reconstruction for all measured metrics in both phantom sizes. The image quality of FBP images is noticeably degraded by noise at lower doses while low dose scans reconstructed with iDose (Miglioretti *et* *al* 2013) and PI have image quality which more closely resemble the highest dose FBP image. This effect is demonstrated visually in figure 4 and confirmed quantitatively by the measured metrics. The results of each metric are represented in figures 5 and 6, and tables A1–A10 show the *t*-test statistics.

All metrics show that iDose^4^ is capable of dose reduction compared to FBP and that PI shows further dose reduction compared to iDose^4^. Furthermore, more aggressive noise reduction, i.e. smoother levels of PI, showed improved performance over less aggressive noise reduction, i.e. sharper levels of PI. When only considering noise and CNR, the different levels of PI achieved dose reduction capabilities between 67%–96% for the small phantom and between 50%–96% for the medium phantom, respectively (figure 5). However, the results of the image similarity metrics RMSE, SSIM, and MSSIM show more conservative dose reduction estimates compared to the estimates obtained from noise and CNR alone. When considering the image similarity metric results, PI demonstrated lower dose reduction capabilities of 25%–83% in the small phantom and 50%–83% in the medium phantom (figure 6). Thus, these image similarity metrics provide additional information about the structural accuracy of the reconstructed images that is not captured by general image quality metrics like noise and CNR.

### 3.2. Phantom size effects

The image quality of the small phantom reconstructions showed an average of approximately 40% improvement across all metrics compared to the matched doses and reconstructions of the medium phantom. Analysis of noise and CNR suggest that there is a slight increase in dose reduction capabilities of PI in the small phantom (67%–96%) compared to the medium phantom (50%–96%). However, the image similarity metrics show the opposite trend, with slightly lower dose reduction capabilities in the small phantom (25%–83%) compared with the medium phantom (50%–83%). Overall, the results from the two phantom sizes showed similar trends in dose reduction.

### 3.3. Summarized potential dose reduction capabilities

**Figure 7.**
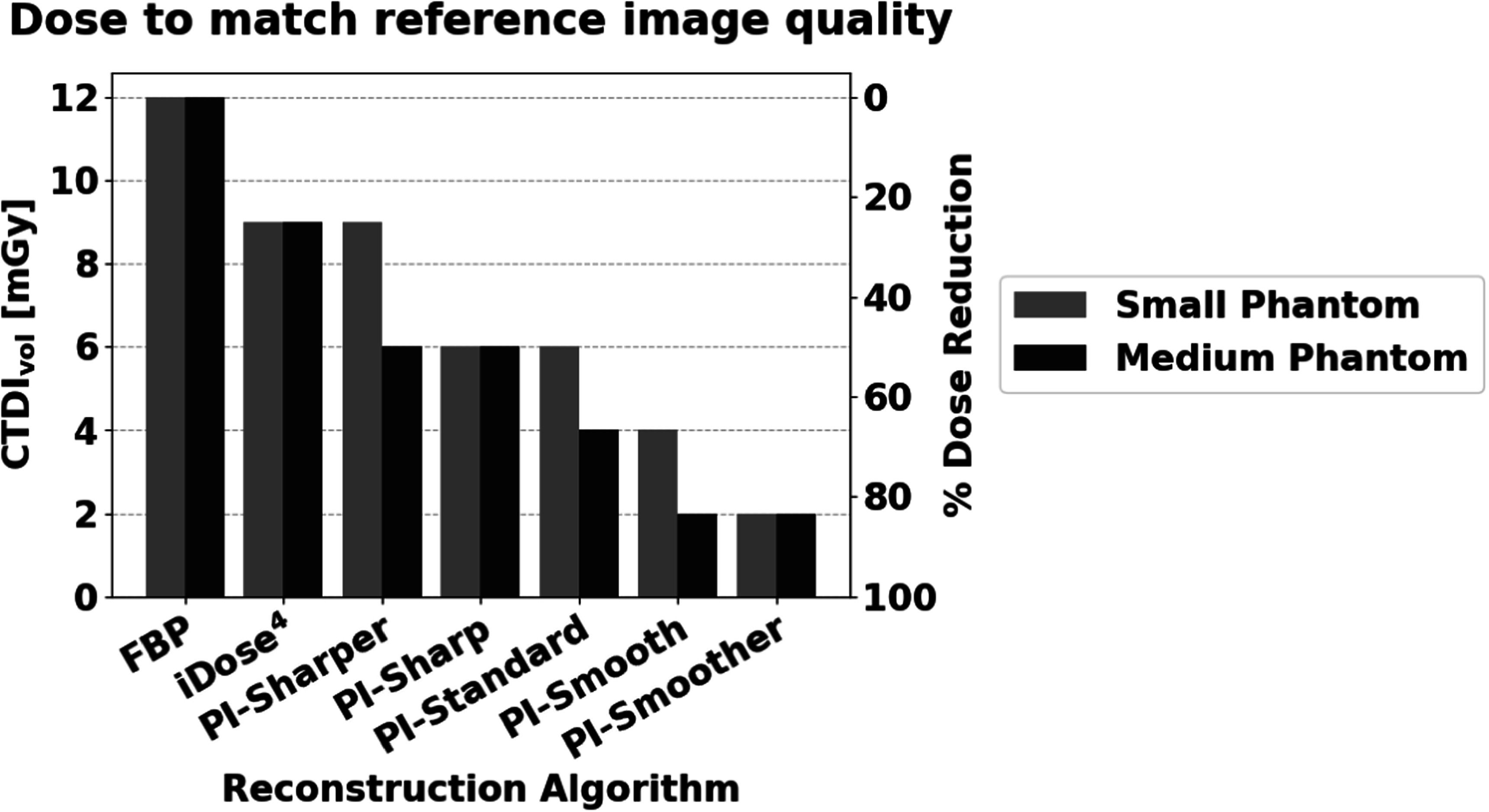
The minimum doses (left axis) required to match or exceed all image quality metrics of the reference images for each reconstruction algorithm, along with the corresponding percent dose reduction (right axis).

The overall dose reduction of each reconstruction algorithm compared to FBP was determined using the minimum dose at which all metrics matched or exceeded the reference performance. These minimum doses are summarized in figure 7 with corresponding dose reduction percentages indicated on the right axis. For both the small phantom and medium phantom, PI demonstrated dose reduction capabilities up to 83% for the highest level of denoising (PI-Smoother).

## 4. Discussion

This study examined the clinical imaging performance of a DLR algorithm, PI, compared to FBP and IR by utilizing a custom-made patient derived PixelPrint lung phantom. The results show that PI is capable of dose reduction for this clinical scenario between 25% and 83% compared to FBP depending on the denoising level of the algorithm and phantom size. This suggests that in some cases PI can produce diagnostic level image quality even for CT scans acquired at lung cancer screening doses of <3 mGy (Kazerooni *et al* 2014). This could mean more effective lung cancer screening and/or reduced radiation burden. Furthermore, these dose reductions are achieved with more natural noise textures compared to IR. The unnatural or ‘plastic’ looking textures in IR images are often attributed to a leftward shift of the NPS curve (Ehman *et al* 2014, Szczykutowicz *et al* 2022). DLR algorithms have been reported to offer varying degrees of improvement in noise texture depending on the specific algorithm assessed and the denoising level used (Szczykutowicz *et al* 2022). A study by Greffier *et al* that looked specifically at the NPS of Standard through Smoother levels of PI showed that the average spatial frequency and peak spatial frequencies of PI were closer to those of FBP compared to iDose^4^, suggesting more favorable noise textures (Greffier *et al* 2023b). These results are complementary to the results presented in the current study.

Despite the differences in study design, this study demonstrated similar trends in noise reduction, image quality improvement, and dose reduction capabilities of PI with previous literature evaluating PI. Greffier *et al* investigated the use of PI for evaluating liver metastases in a patient study (Greffier *et al* 2023a) which demonstrated that more aggressive levels of denoising for PI (Smooth and Smoother) resulted in better performance in the lowest dose scans, as shown in this study. In a separate study examining the use of PI in chest imaging, Greffier *et al* reported dose reductions of 58% and 83% compared to iDose^4^ Level 4 for PI-Smooth and PI-Smoother respectively, based on task-based image quality assessment of simulated ground glass opacifications in a standard, geometric phantom (Greffier *et al* 2022a). Similarly, our study results show that for a medium-sized phantom, if the results from MS SSIM are excluded, PI-Smooth and PI-Smoother have a 58% and 88% dose reduction potential respectively compared to iDose^4^ level 3. However, when MS SSIM is included the dose reduction potential becomes more conservative. The correlation between our results and previously published literature help to validate the use of PixelPrint phantoms in the evaluation of DLR.

Studies evaluating other DLR algorithms have also demonstrated reduced noise and improved lesion detectability in DLR compared to IR or FBP (Son *et al* 2022, Miyata *et al* 2022, Park *et al* 2022, Greffier *et al* 2022b, Koetzier *et al* 2023). The exact percentage of dose reduction reported was heavily dependent upon many factors including the clinical scenario, the reference dose, reference reconstruction algorithm, DLR algorithm and denoising strength, and specific metrics evaluated. Overall, the results from studies of several other reconstruction algorithms included a wide range of dose reduction estimates between 30% and 85%, which is similar to the results for PI.

Furthermore, this study demonstrates the additional information which evaluations using PixelPrint phantoms can provide compared to patients and standard phantoms. Unlike in patient studies, an evaluation using PixelPrint phantoms allows for the comparison of a wide range of doses beyond the dose range typically acceptable in clinical practice. Thus, ground truth data can be obtained by acquiring a higher than standard radiation dose. Additionally, the dose reduction capability of DLR can be probed more precisely by repeatedly acquiring increasingly lower dose data and comparing the resultant images directly. The ability to acquire images without any patient motion between scans facilitates comparison between images via similarity metrics such as RMSE.

Compared to traditional CT phantoms, the presence of clinically relevant structures and details in PixelPrint phantoms is advantageous because it enables comparison of reconstruction accuracy for complex structures. Reconstruction accuracy can be evaluated by using image similarity metrics such as SSIM to compare a reconstructed image to the selected ground truth. The inclusion of these image similarity metrics resulted in more conservative dose reduction estimates compared to the results obtained using only general image quality metrics such as noise and CNR. This may be because although the DLR algorithm can essentially tune noise levels to almost any desired amount, some information from detailed structures may not be recoverable. As a result, analyzes on non-clinical structures such as those found in traditional CT phantoms cannot adequately capture these algorithms’ diagnostic imaging performance. PixelPrint phantoms can also provide additional information about patient size dependency in DLR dose reduction. A previous patient study using general image quality metrics showed that iDose^4^ achieved higher dose reduction in smaller patients versus larger patients (Arapakis *et al* 2014). In the present study, only considering the noise and CNR measurements results in the same trend while the inclusion of image similarity metrics results in a reversal of the trend such that the small phantom size has slightly reduced dose reduction potential. Finally, it has been reported that a possible concern with DLR is that if certain lesions are not well represented in training sets, these lesions may not be reconstructed accurately in DLR images (Nagayama *et al* 2021). This is not something that can be tested with standard geometric phantoms but can be easily investigated using different PixelPrint phantoms with various lesions and known ground truth images. These findings suggest that the use of PixelPrint phantoms in conjunction with image similarity metrics provides valuable information which is not available from standard phantoms or patient studies alone for determining dose reduction capability.

The present study has a few limitations. First, PI was compared to only one denoising level of iDose^4^, the default level for lung imaging. To form a more robust understanding of the improvement that PI affords over IR, it would be valuable to compare PI to more denoising levels of iDose (Miglioretti *et al* 2013). Similarly, the same phantom could be used to evaluate and compare multiple DLR algorithms including both commercial and open-source algorithms. Second, this study only utilized one phantom and thus only one example of patient anatomy. Future studies involving more phantoms from different patients could improve our insights into the behavior of PI in different clinical scenarios and disease states. This could be especially useful in rarer or more unique clinical cases where patient data is limited. Third, this study does not include a reader study with subjective image quality scores. However, there are other existing studies that include a reader study (Greffier *et al* 2022a, 2023b, Philips Healthcare 2024) and those results show good alignment with the results of the present study. Fourth, the HU range of the phantom used in this study is limited to between −867 and 115 HU for the PLA material used. Other printing materials are being investigated in order to increase the HU range of PixelPrint phantoms (Mei *et al* 2022a). Finally, the raw projection data corresponding to the patient images used to create the PixelPrint phantom was unavailable, preventing a direct comparison between PI performance on phantom data and its performance on the source patient data.

## 5. Conclusion

This study demonstrates the dose reduction capabilities of a DLR algorithm, Precise Image, in the context of lung imaging with GGOs. For this clinical scenario, PI has the capability of producing diagnostic image quality at up to 83% lower radiation dose, even surpassing the dose reduction capabilities of iterative reconstruction. These results are consistent with existing literature evaluating DLR. Images reconstructed using PI demonstrate not only improved noise and contrast compared to FBP and iterative reconstruction, but also improved structural accuracy of lung features such as GGO lesions. The use of PI can improve the clinical utility and viability of lower dose CT scans, ultimately improving patient care while reducing radiation exposure.

The PixelPrint phantom used in this study offers an improved testing environment with more realistic tissue structures and attenuation profiles compared to other CT phantoms. This is particularly important for the evaluation of nonlinear reconstruction algorithms such as DLR. Thus, PixelPrint phantoms can elevate the clinical relevance of phantom evaluations of new and emerging CT technologies, which will lead to more rapid translation of these technologies into medical practice.

## Figures and Tables

**Figure A1.**
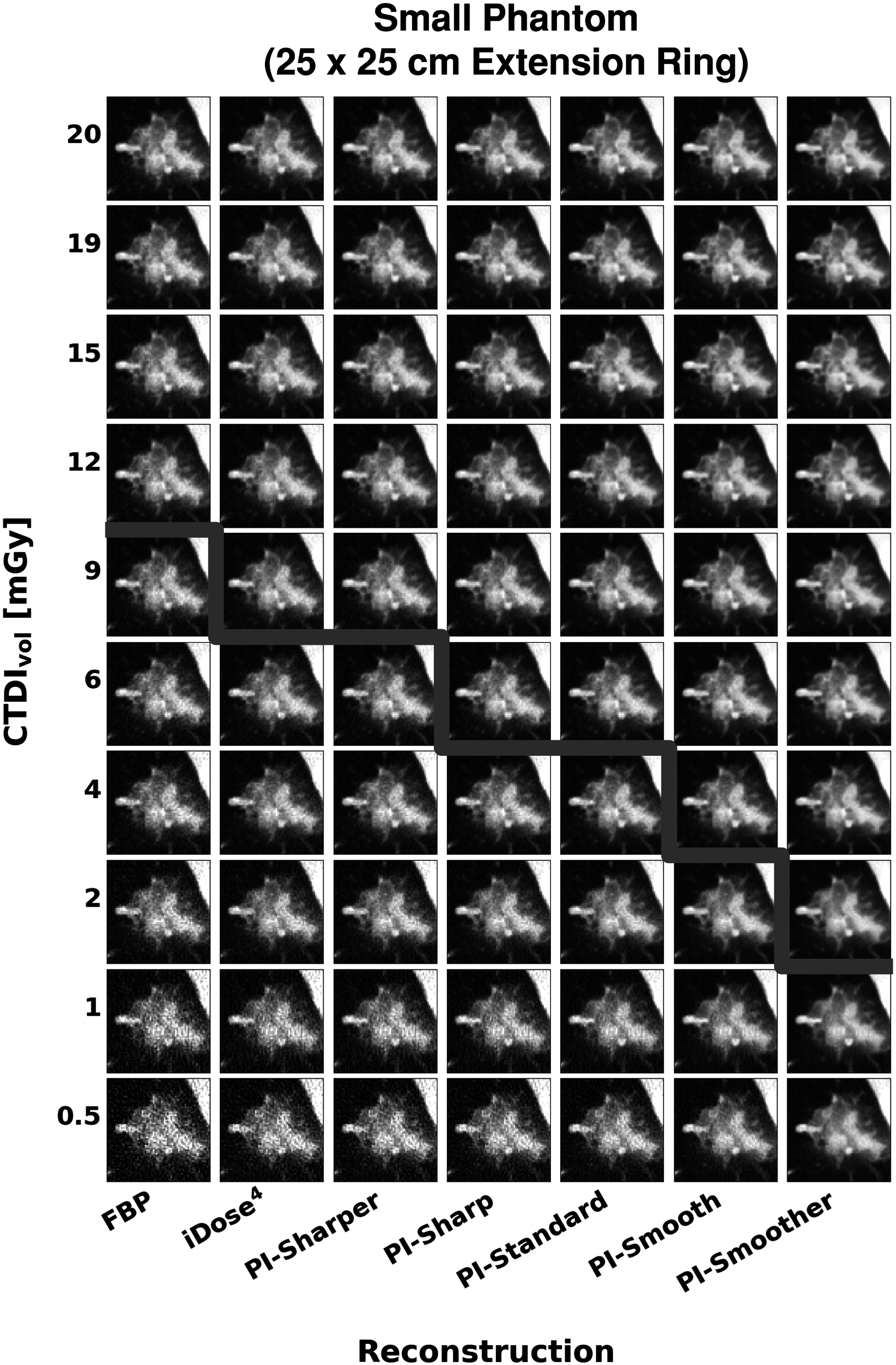
Images of the GGO lesion taken from the small phantom at each dose and reconstruction combination. WL: −500, WW: 1000 HU. Images above the blue line match or exceed the diagnostic reference level (12 mGy) image quality for all measured metrics.

**Figure A2.**
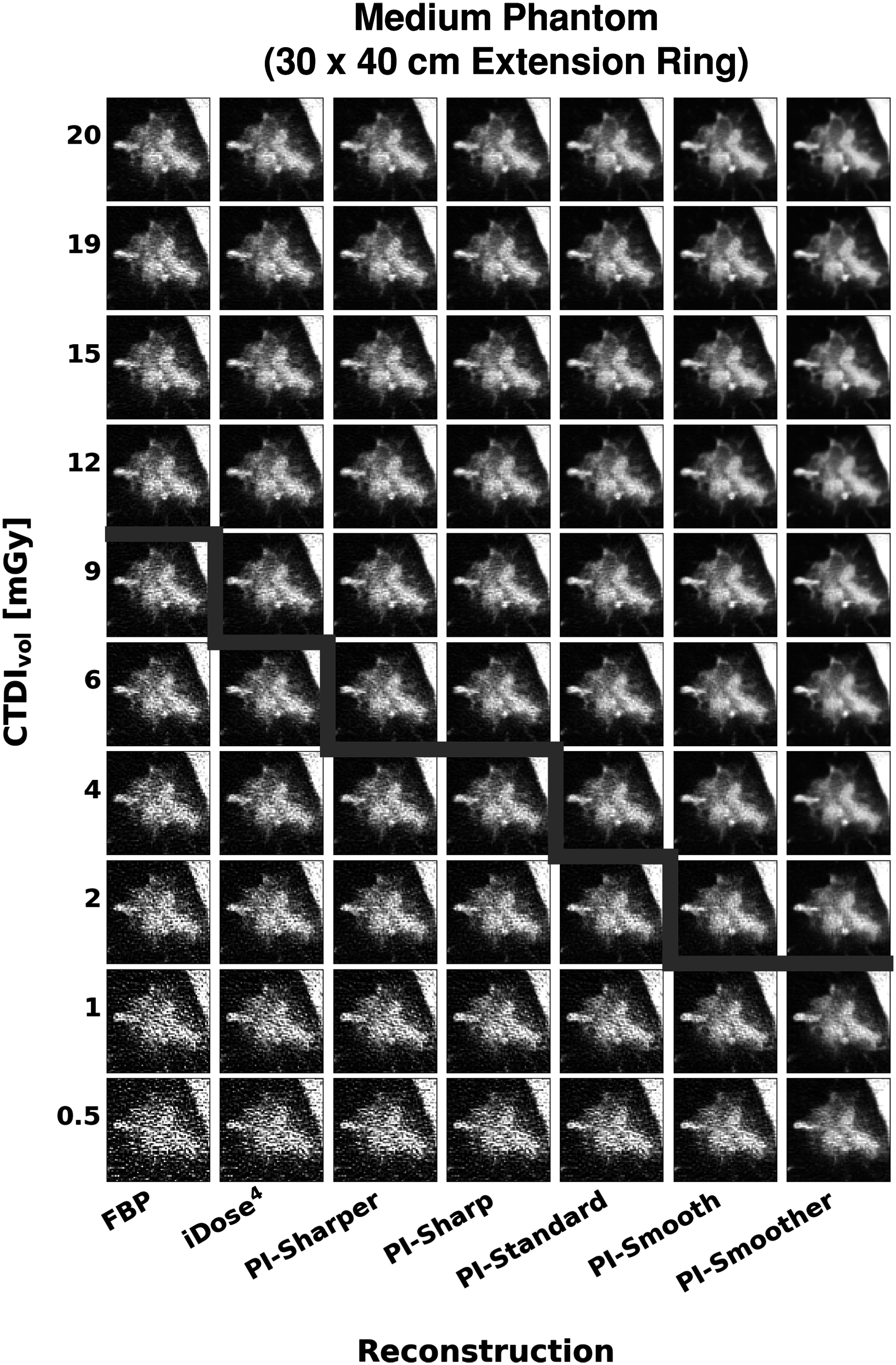
Images of the GGO lesion taken from the medium phantom at each dose and reconstruction combination. WL: −500, WW: 1000 HU. Images above the blue line match or exceed the diagnostic reference level (12 mGy) image quality for all measured metrics.

## Data Availability

The data cannot be made publicly available upon publication because they are not available in a format that is sufficiently accessible or reusable by other researchers. The data that support the findings of this study are available upon reasonable request from the authors.

## Author contributions

Conception: JI, SH, PN, Design: JI, SH, KM, AP, LR, PN, Data acquisition: SH, EW, Analysis: JI, Drafting: JI, Revising: JI, SH, KM, AP, OS, LL, GG, PN.

## Appendix

Tables A1–A10. *t*-values and *p*-values obtained from the two-sample, one-tailed student’s t-test for each image metric calculated. The cells highlighted with red indicate where the *t*-value signifies worse performance (*t* < 0 for Noise and RMSE, *t* > 0 for CNR, SSIM, and MS SSIM) than the reference (FBP, 12 mGy). The cells highlighted in blue indicate where the *p*-value suggests a statistically significant result (*p* < 0.01). Cells above the thick black line have performance that is better than or not statistically different than the reference (*t* > 0 and/or *p* > 0.01). Cells below the thick black line have performance that is statistically worse than the reference (*t* < 0 and *p* < 0.01).

**Table A1.** Noise—small phantom.

**Table A2.** CNR—small phantom.

**Table A3.** RMSE—small phantom.

**Table A4.** SSIM—small phantom.

**Table A5.** MS SSIM—small phantom.

**Table A6.** Noise— medium phantom.

**Table A7.** CNR—medium phantom.

**Table A8.** RMSE—medium phantom.

**Table A9.** SSIM—medium phantom.

**Table A10.** MS SSIM—medium phantom.
